# Investigation of VOCs associated with different characteristics of breast cancer cells

**DOI:** 10.1038/srep13246

**Published:** 2015-08-25

**Authors:** Luca Lavra, Alexandro Catini, Alessandra Ulivieri, Rosamaria Capuano, Leila Baghernajad Salehi, Salvatore Sciacchitano, Armando Bartolazzi, Sara Nardis, Roberto Paolesse, Eugenio Martinelli, Corrado Di Natale

**Affiliations:** 1 Labotatory of Biomedical Research “Fondazione Niccolò Cusano per la Ricerca Medico-Scientifica”, Niccolò Cusano University, Rome, Italy; 2 Department of Electronic Engineering, University of Rome Tor Vergata, Via del Politecnico 1, 00133 Rome, Italy; 3 Department of Clinical and Molecular Medicine, University of Rome “Sapienza”, Rome, Italy; 4 Department of Pathology, Universitary Hospital Sant’Andrea, Rome, Italy; 5 Department of Oncology-Pathology, Cancer Center Karolinska, Karolinska Hospital, Stockholm, Sweden; 6 Department of Chemical science and technology, University of Rome Tor Vergata, Via di Tor Vergata, 00133 Rome, Italy

## Abstract

The efficacy of breath volatile organic compounds (VOCs) analysis for the screening of patients bearing breast cancer lesions has been demonstrated by using gas chromatography and artificial olfactory systems. On the other hand, *in-vitro* studies suggest that VOCs detection could also give important indications regarding molecular and tumorigenic characteristics of tumor cells. Aim of this study was to analyze VOCs in the headspace of breast cancer cell lines in order to ascertain the potentiality of VOCs signatures in giving information about these cells and set-up a new sensor system able to detect breast tumor-associated VOCs. We identified by Gas Chromatography-Mass Spectrometry analysis a VOCs signature that discriminates breast cancer cells for: i) transformed condition; ii) cell doubling time (CDT); iii) Estrogen and Progesterone Receptors (ER, PgR) expression, and HER2 overexpression. Moreover, the signals obtained from a temperature modulated metal oxide semiconductor gas sensor can be classified in order to recognize VOCs signatures associated with breast cancer cells, CDT and ER expression. Our results demonstrate that VOCs analysis could give clinically relevant information about proliferative and molecular features of breast cancer cells and pose the basis for the optimization of a low-cost diagnostic device to be used for tumors characterization.

Breast tumor is the first cause of death for cancer in woman worldwide^1^. Chance of cure improves considerably if the disease is diagnosed at an early stage when the tumor is still localized and asymptomatic^2^. Breast cancer early detection therefore is mainly based on clinical examination and imaging performed by mammography, ultrasound and nuclear magnetic resonance. Mammography and ultrasound are the most commonly imaging tools used for the detection and characterization of breast abnormalities. However, both techniques are hampered by relatively low sensitivity and specificity and may expose patients to over-diagnosis and over-treatment of benign lesions or missed diagnosis and failure to treat cancerous lesions. In addition, personalized cancer treatments require a complex invasive and time-consuming analysis of many different parameters, such as histological type and grading, evaluation of Estrogen Receptor (ER), Progesterone Receptor (PgR), HER2 and Ki67 protein expression by immunohistochemistry (IHC), gene mutation analysis by DNA sequencing and chromosomal alterations by Fluorescence *In Situ* Hybridization (FISH)^3^.

Taken together all these aspects stimulate the research of new diagnostic tools that could promptly support the clinicians in breast cancer treatment decision-making.

Among them, the analysis of volatile organic compounds (VOCs) emerges as a new frontier for cancer diagnosis in particular because it is non-invasive and potentially inexpensive. The concept has been demonstrated with different types of cancer^4^,^5^,^6^,^7^,^8^,^9^,^10^,^11^,^12^,^13^,^14^,^15^,^16^,^17^,^18^,^19^,^20^,^21^,^22^,^23^,^24^. In particular, the diagnostic accuracy of VOCs detected in the breath of patients bearing breast cancer lesions has been extensively analyzed^5^,^6^,^7^,^8^,^9^,^10^,^11^,^12^,^13^,^14^,^15^.

Breath analysis could be applied in the early identification of tumors, but in order to provide histological and molecular information useful to clinicians for the selection of the specific treatment of a neoplastic lesion, VOCs analysis should be applied directly to tumor.

To this regard, *in vitro* experiments demonstrate that artificial olfactory systems can discriminate between tumor and normal cell lines and also among tumors-derived cells bearing different molecular alterations^16^,^17^,^18^,^19^,^20^,^21^. The sensitivity of this technique is also sufficient to follow the evolution of cancer as shown in murine models for melanoma and lung cancer^22^. However, only few studies have been addressed to the analysis of the VOCs released in the headspace of breast cancer-derived cell lines to increase the comprehension of the metabolic alterations associated with breast cancer transformation and enable the development of cost-effective and non-invasive breast cancer diagnostic tools^24^.

In this study we searched with GC-MS for VOCs signatures that can be associated to the headspace of culture medium of tumor breast cell lines and that can signal the occurrence of specific breast cancer prognostic markers.

The analysis of GC-MS data shows that the abundance of 13 VOCs changes between different breast cancer cell lines and is sensitive to the cell doubling time (CDT) and to the expression of specific prognostic factors such as Estrogen Receptor (ER), Progesterone Receptor (PgR) and HER2.

The same samples were also measured with a novel approach that considers a metal-oxide semiconductor gas sensor operated in a thermal modulation condition. Actually this solution differs from other approaches that consider a sensor arrays working at room temperature^13^,^19^,^20^,^21^,^22^ exploiting the additional information obtained by a single sensor operating at different temperatures to compensate the lack of further sensors^25^,^26^,^27^.

A statistical analysis of the sensor signals results in a good classification of the breast cell lines based on transformed condition, replication time and ER expression. All these results confirm that the volatile compounds might provide a cost-effective diagnostic tool useful for the characterization of the breast tumor lesion aimed at the optimization of the therapeutic treatment.

## Results

### Discrimination of the breast cancer cell lines characteristic by GC-MS analysis

**Figure 1:**
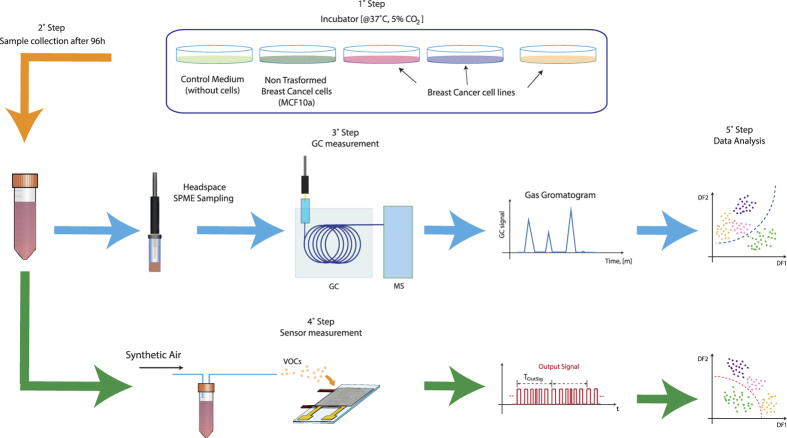
Schematic of the experimental measurements.

The VOCs associated with breast cell metabolism have been analyzed with GC-MS by comparing culture medium headspace of six breast cell lines with medium without cells (Fig. 1, Table 1).

**Figure 2:**
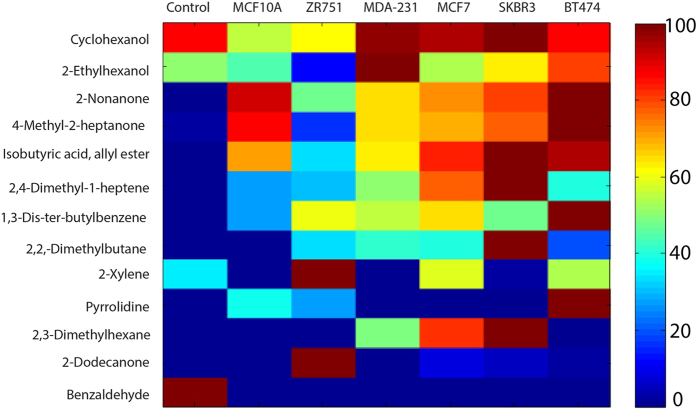
Fingerprint patterns of VOCs detected in headspace of breast-derived cell lines. Heat-map with all the selected VOCs from culture media headspace. Color-coding shows the abundance of each compound measured in the sample normalized to the maximum abundance calculated in all samples.

After a preliminary phase (see material and methods), the abundances of 13 selected VOCs have been correlated with the different cell lines showing for each of them specific patterns (Fig. 2). Collectively these VOCs significantly discriminate empty control medium from medium exposed to cells (Manova p = 1.6 ×1 0^−16^) (Table 2). The detected compounds belong to the classes of hydrocarbons, ketons, alcohols, aldehydes, amine aromatic and carboxylic acid (Table 2).

Among the 13 VOCs identified, the abundance of nine of them significantly increases in the headspace of samples related to breast cell lines (Table 2). Eight of them are absent in control medium samples, indicating a specific association of these compounds with cell metabolism (Fig. 2). Benzaldehyde is the only compound that was exclusively found in the control (Fig. 2, Table 2). For this reason, this compound has been excluded in the analyses aimed at studying the properties of breast cell lines.

Altogether, the 13 VOCs identified in previous analysis are also statistically meaningful for the discrimination between non-transformed and tumor-derived cell lines (p = 7.7 × 10^−3^) (Table 3). To identify the specific VOCs related with breast cancer cells, the amount of each compound was compared between breast non transformed cell (MCF-10A) and cancer cell lines, evidencing a list of eight specific compounds (Table 3). The VOCs whose abundance significantly increases in cancer cells are four hydrocarbons (2,4-Dimethyl-1-heptene, 2-Xylene, 2,3-Dimethylhexane, 2,2-Dimethylbutane), one secondary alcohol (Cyclohexanol) and one ketone (2-Dodecanone). A decrease of abundance is observed for two ketones (2-Nonanone and 4-Methyl-2-heptanone). These results indicated that a specific VOCs signature characterizes the headspaces of breast tumor-derived cell lines.

A further step in the study was to investigate the relationship between the VOCs and some cancer cell features such as the growth rate and the expression of specific proteins. In particular, the cell doubling time (CDT) and the expression of the three main prognostic markers of breast cancer, such as ER, PgR and HER2, have been considered (Table 1, Supplementary figure 1).

MANOVA analysis shows that the same VOCs, previously identified by GC-MS analysis (Fig. 2 and Table 2), discriminate also the cancer cells between long (major than 48 h) and short (less or equal to 48 h) CDT (p = 5 × 10^−4^), ER positive from ER negative (p = 3.5 × 10^−5^), PgR positive from PgR negative (p = 7.4 × 10^−4^) and HER2 overexpressing cells (p = 0.03) (Table 4).

Considering the behavior of each compound, we observed that seven VOCs showed significant differences between high and low replicating breast cells. Six of them were more abundant in cells with short CDT (2,4-Dimethyl-1-heptene, 2,3-Dimethylhexane, Cyclohexanol, 2-Ethylhexanol, Isobutyric acid, allyl ester, 4-Methyl-2-heptanone) and 2-Dodecanone only in cells with long CDT (Table 4).

The abundance of five compounds was significantly different between ER negative and ER positive cell lines, four among PgR negative and PgR positive cells and, one in HER2 overexpressing cell lines (Table 4). Interestingly, both CDT and marker receptors correlated with the abundance of 2-Dodecanone. Moreover, 2-Xylene, 2-Ethylhexanol and 2-Dodecanone show the same behavior in the headspaces of both ER and PgR positive cell lines (Table 4) suggesting that the same VOCs could be associated with specific receptor-related metabolic pathways in these cells.

These results suggested that the identified VOCs signature could furnish information regarding the replication rate and the expression of breast cancer prognostic molecular markers.

### Discrimination of the breast cancer cell lines characteristic by metal-oxide semiconductor gas sensor analysis

Chemical sensors are expected to provide a full exploitation of the GC-MS findings in order to develop effective diagnostics tools^28^.

**Figure 3:**
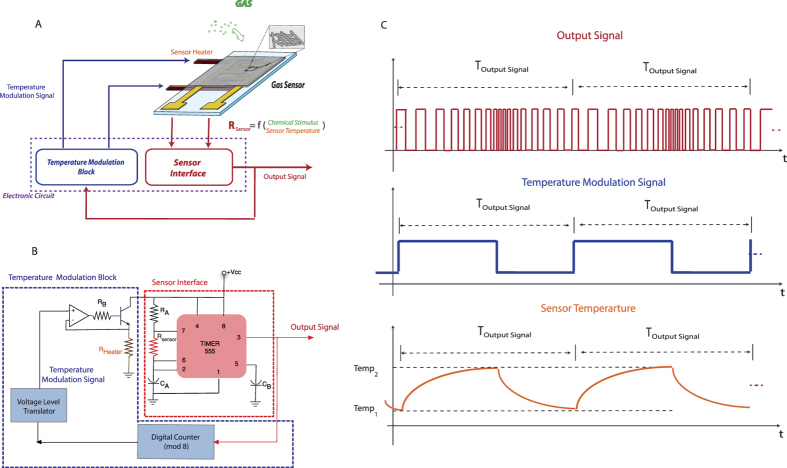
(**A**) Schematic of the self-modulation sensor. (**B**) Circuit implementation used in this work; (**C**) From top to bottom, examples of output signal, temperature modulation signal and sensor temperature.

In this paper we investigated the discrimination properties of metal-oxide semiconductor gas sensor operated in self-temperature modulation mode as described in Fig. 3. This approach allows to overcome the limitation of using a single sensor in a complex discrimination task exploiting the increase of the information content given by a temperature modulation. Preliminary results put in evidence the potentialities of the self-temperature modulation in the classification of different volatile compounds^27^.

**Figure 4:**
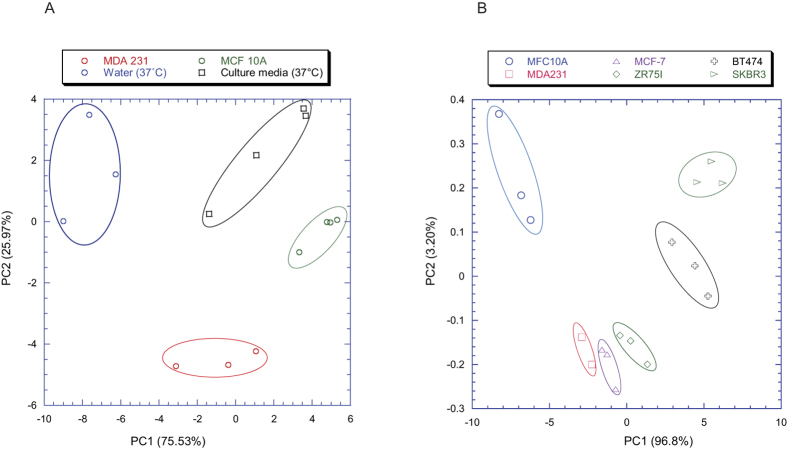
Clustering relationship of self-modulated sensor outputs. (**A**) Score plots of the first two principal components built with sensor data. A clear separation is observed among the MCF10A non-transformed control cell line (encircled in green), MDA231 cancer cell line (encircled in red), culture media without cells (encircled in black) and distilled water (encircled in blue). (**B**) Score plots of the first two principal components built with sensor data. A comparison of the sensor responses for the MCF-10A breast control cell line and five breast cancer cell lines (MDA231, MCF-7, ZR751, BT474, SKBR3) is shown.

A first set of experiments was aimed at distinguishing among the headspaces of the culture media of MCF-10A control cell line, MDA231 breast cancer cell line, culture media without cells and distilled water. The discrimination among the four above-mentioned classes is shown by the scores plot of the first two principal components of the PCA model built with the features of the thermal modulation profile (Fig. 4A).

In order to characterize the specific VOCs fingerprint of breast control and cancer cell lines, the analysis was extended to all the breast cell models. The PCA scores plot in Fig. 4B shows a difference between the signals from breast non-transformed MCF-10A cells and breast cancer cell lines. Interestingly, breast cancer cell lines cluster in two major groups (SKBR3 and BT474 in the first, MDA231, MCF7 and ZR751 in the second one) notably separated in both components (Fig. 4B). A PLS-DA classification model aimed at identifying the cancer cell (cancer cell vs. not transformed cell and culture media) achieved about 85% of correct classification (accuracy) with a sensitivity and specificity equal to 88% and 80% respectively (Table 5). This result is particularly interesting since it is obtained with a single sensor instead as usual with a sensor array.

A PLS-DA model was calculated from the sensor signals in order to discriminate the breast cancer cells according to the cell features previously discussed (Table 1).

The PLS-DA model shows that the sensorial system discriminates with high accuracy breast cell lines with low CDT (classification rate of 88%, sensitivity of 89% and specificity of 87%) and positive for ER expression (classification rate of 85%, sensitivity of 86% and specificity of 83%) (Table 6A and B). PgR expression and HER2 overexpression are classified with accuracy lower than 75% (data non shown).

## Discussion

The analysis of VOCs provides an elegant alternative approach to cancer diagnosis. However, the practical use is still limited by a lack of validated cancer-derived metabolites, and by a consequent lack of sensing technologies optimized to their detection. In this work we have analyzed the VOCs emitted by the culture media of different breast cancer cell lines with the GC-MS and a temperature modulated metal oxide gas sensor highlighting the potentialities of this approach to obtain diagnostic information with high impact in the clinical management of breast cancer patients.

As demonstrated in *in vivo* and *in vitro* studies, specific VOC signatures are associated with the presence of neoplastic lesions and with the molecular alterations in oncogenes and tumor suppressor genes in tumor cells^20^,^21^. Any rapid and cost-effective prognostic and therapeutic molecular information about a tumor obtained with this technique, might have a dramatic impact on clinical management of tumor patients and open new insight in the diagnostic potential of VOC signatures.

Different studies analyzed the potentiality of sensors for the screening of breast cancer patients by breath VOCs analysis^5^,^6^,^7^,^8^,^9^,^10^,^11^,^12^,^13^,^14^,^15^. However, breath analysis may not be an optimal approach to detect the VOCs fingerprint associated to specific molecular alterations of cancer cells. This because of the low abundances of the key compounds and the interfering effects of non-cancer related VOCs present in the exhaled air.

In our study we analyzed the specific odor print of breast cancer cells by analyzing a simpler and less contaminated sample: the headspace of conditioned culture medium of cell lines plated in proliferative conditions.

This kind of sample allows detecting specific VOC exchanges that take place during normal and tumor breast cell proliferation and could be also considered an *in vitro* model for VOCs modifications occurring *in vivo* between tumor cells and body fluids, like blood, interstitial or lymphatic fluids.

We demonstrated, by GC-MS analysis, that a pattern of 13 VOCs discriminates the headspace of breast cell lines growth media. Similar VOC patterns were reported for other cell lines^29^. In particular, the increase in 2,4-Dimethylheptene, 4-Methyl-2-heptanone, 2-Nonanone, 2-Ethylhexanol, 2,3-Dimethylhexane and the decrease of Benzaldehyde have been also observed in lung and hepatocellular carcinoma derived cell lines and in human fibroblast^16^,^30^,^31^. It is interesting to note that 2-Nonanone is the most sensitive compound. It derives from Nonane metabolism by the enzymatic activity of cytochrome P450 and its increase could be associated with the high activity of the different isoforms of this enzyme observed in breast-derived cells^32^,^33^,^34^.

The remaining compounds are never been previously observed in *in vitro* studies. This could be due to the specific cell lines analyzed in this study and the peculiar culture conditions used in our experiments. In fact, in contrast to the conditions used in previous studies, we plated our cell lines at low densities in order to collect, during the incubation time, all compounds exchanged in the proliferative phase.

The identified set of VOCs is also able to significantly discriminate breast cancer cells suggesting that non-transformed and tumor cell lines could be characterized by a specific VOCs signature by which it is possible differentiate them, as also recently demonstrated by He *et al.* by using different technical and methodological conditions^24^.

Among compounds increased in tumor breast cancer cells the more represented classes are the hydrocarbons, and some of these (2,4-Dimethylheptene, 1,3-Di-tert-butylbenzene, and 2-Xylene) have been previously detected in breath samples of breast and lung cancer patients^6^,^7^,^35^. Previous reports of breath biomarkers associated with breast cancer patients identified hydrocarbons, in particular alkanes and alkane derivatives (methyl-alkanes), as main VOCs breast cancer biomarkers^6^,^7^,^8^,^9^,^10^,^11^,^12^,^13^,^14^,^15^.

This enhanced production of alkanes has been supposed to be due to the increased oxidative stress correlated with tumor progression^36^. Gene and/or protein changes and increased metabolism that accompanied tumor cell proliferation lead to oxygen free radical production and peroxidation of polyunsaturated fatty acids in membranes and, hence, to the emission of alkanes and methyl-alkanes.

As reported for other types of cancer cells, our results demonstrate that breast tumor cells can be classified by specific VOCs signatures, providing the rationale for the set-up of technologies aimed to the detection of these biomarkers.

To this purpose, we tested the performances of a commercial metal-oxide semiconductor gas sensor to discriminate the VOCs patterns released in the headspaces of culture medium of breast-derived cell lines. Results highlight that from the sensor data we can classify with high accuracy cancer-derived samples. Despite the small number of non-transformed and tumor breast cell models analyzed, our results encourage the application of this kind of sensors for these applications. It is straightforward that the system performance can be further improved increasing the number and the kinds of sensors.

Recent *in vitro* studies provided evidences for the existence of VOCs signatures characteristic of genetic mutations associated with lung cancer cells or with the metastatic potential of hepatocarcinoma cell lines^20^,^21^,^37^. As a consequence, we analyzed our data to evidence the influence of cancer cell indicators such as the CDT and the expression of the most important diagnostic and prognostic immunohistochemical markers used for the characterization of breast cancer biopsy, namely ER, PgR and HER2.

This analysis demonstrated that CDT elicits changes in the VOCs profile that can be measured both with the GC-MS and the gas sensor. These interesting preliminary evidences of the correlation of VOCs with cancer cell proliferation necessitate of further experiments to fully exploit VOCs as proliferation markers of cancer cells.

The same analysis of GC-MS data also demonstrated the relationship between the VOCs profiles and the molecular expression of ER, PgR and HER2. Gas sensor data discriminate between cell lines with different ER molecular status but low discrimination accuracy has been observed with respect to PgR and HER2 molecular status. This is likely due to the small difference in the VOCs signature identified by GC-MS analysis among PgR and HER2 positive and negative cells (Table 5).

In this study we performed our analysis using *in vitro* breast cancer cell line models to discriminate the VOCs specifically emitted by tumor cells, without interferences from other cell types that are normally present in a tumor lesion. Actually the main scope of the work is not related to early diagnosis of the tumor lesion but to support the medical staff in the choice of the best therapeutic treatment of specific kind of cancer. However, a validation on biological specimens (e.g. blood, interstitial or lymphatic fluids, cytological samples) obtained from breast cancer patients will be performed in a future investigation to understand the diagnostic potentials of the presented results.

## Conclusion

In this paper a study about the potentialities of the associated VOCs of breast tumor cell lines to identify new proliferative and molecular biomarkers has been presented. To this regard the headspace of the culture media of cancer cell lines has been analyzed with the GC-MS and with a gas sensor operated under thermal modulation. The GC-MS has evidenced a list of potential VOCs whose contemporaneous presence is correlated with specific characteristics of the breast cancer cells. The same characteristics can also be captured by a simple gas sensor. The promising results obtained with a commercial sensor foreshadow the possibilities to improve the system performances optimizing the sensor selection or considering an ensemble of sensors. Nevertheless, further *in vitro* and *in vivo* studies using many other cell models are necessary to validate the VOCs pattern and to optimize the sensor system for a future extension to clinical tests.

## Material and Methods

### Cell culture and immunocytohistochemistry

Six human breast cell lines were used: MDA-231, MCF-7, SKBR3 (kindly provided by Prof. Giannini G., Department of Molecular Medicine, “Sapienza” University of Rome, Rome, Italy), BT474, ZR75-1 and MCF-10A (kindly supplied by Dr. Falcioni R., Department of Experimental Oncology, Regina Elena National Cancer Institute, Rome, Italy)^38^,^39^,^40^,^41^. Cell lines have been propagated under the conditions suggested by the supplier in order to preserve their characteristics after *in vitro* passages. The immortalized, non-transformed human mammary epithelial cell line MCF-10A was grown in DMEM/F12 medium (Sigma-Aldrich) supplemented with 5% fetal bovine serum, 20 ng/ml epidermal growth factor (EGF), 10 μg/ml insulin, 0.5 μg/ml hydrocortisone (Sigma-Aldrich), 100 units/ml penicillin and 100 μg/ml streptomycin (Sigma-Aldrich), as previously described^42^. The five human breast cancer-derived cell lines MDA-MB-231, MCF-7, SKBR3, BT474 and ZR75-1 were grown in DMEM high-glucose medium (Sigma-Aldrich) supplemented with 10% fetal bovine serum (Sigma-Aldrich), 100 units/ml penicillin and 100 μg/ml streptomycin (Sigma-Aldrich). All cell lines were cultured under standard conditions at 37 °C in humidified atmosphere containing 5% of CO_2_. The CDT of the different cell lines in the experimental culture conditions has been evaluated by cell count (Table 1). A doubling time major than 48 hours has been considered as low replication rate. The CDT of MCF-10A cells was also analyzed in incubation culture medium (DMEM), used in VOCs analysis, to asses eventual alteration of growth rate, and no significant changes were observed up to 96 h of culture (data not shown). The molecular status of Estrogen Receptor (ER), Progesterone Receptor (PgR) and HER2 in each cell line was analized by immunocytohistochemistry (IHC). For each cell line cell block preparation and IHC were done as previously described^43^ by using the following monoclonal mouse anti-Human anti-ER (Clone 1D5) and anti PgR (Clone 636) antibodies (DakoCytomation, Glostrup, Denmark). HER2 expression was assessed by using the HercepTest™ for Dako Autostainer (DakoCytomation, Glostrup, Denmark). IHCs were assessed by one experienced pathologists (Supplementary figure 1).

For VOCs analysis, each breast-derived cell line was seeded in six separated culture flasks (25 cm^2^) with 5 mL of its specific culture medium for 24 h. The number of plated cells was chosen based on the specific doubling time of each cell line, in order to maintain each cell lines in a proliferative phase and to obtain a comparable cell number at the end of the incubations.

After 24 h, the specific culture medium was removed and replaced with 5 mL of the DMEM culture medium. Cells were grown in these conditions for 96 hrs, up to a confluence of 50%-60% (around 1.5 × 10^6 ^cells/flask). After this incubation period, the DMEM culture medium was harvested, centrifuged at 1200 rpm for 5 min to remove detached cells, and collected in sterilized glass vials. At the end of the incubation, cell number and viability were evaluated respectively by cell count and Trypan Blue exclusion, in order to control cell density in all flasks and assess the effect of any cell stress during the incubation time. The *control medium* was obtained by incubating DMEM culture medium in the same conditions as the cell samples, but without seeded cells. The experimental set-up used for the cell culture headspace analysis is shown in Fig. 1.

### Solid Phase Micro-Extraction (SPME) headspace sampling

A volume of 5 ml of the sample (control or conditioned medium) was closed in a 20 ml vial (Flat bottom headspace vial, SUPELCO, Bellefonte, PA, USA) sealed with a PTFE/Silicone crimp seal (SUPELCO, Bellefonte, PA, USA) and stored at 4 °C until the analysis.

Volatile organic compounds composing sample headspace were pre-concentrated onto a SPME fiber coated with 50/30 μm Divinylbenzene/Carboxen/PDMS (SUPELCO, Bellefonte, PA, USA). Before each analysis session the fibers were conditioned at 270 °C for 1 h.

Filled vials were placed in a water bath equilibrated at 40 °C. SPME fiber was then manually exposed to sample headspace for 1 h.

The fiber with sampled VOCs was transferred to the GC–MS and desorbed at the injection port of the GC with an inlet temperature of 250 °C for 3 minutes. The analyses were conducted in the same day of the sample collection.

### Gas Chromatography–Mass Spectrometry (GC-MS)

The analyses of SPME sampled VOCs were performed with a GCMS-QP 2010 Shimadzu series Gas Chromatograph Mass Spectrometer, equipped with EQUITY-5 (poly(5% diphenyl/95% dimethyl siloxane) phase, SUPELCO, Bellefonte, PA, USA) capillary column, 30 m length × 0.25 mm I.D. × 0.25 μm thickness, and conducted in split-less mode using ultra-high purity helium as carrier gas. The instrument was controlled in linear velocity. Pressure was 24.9 kPa, flow parameters were 5.9 ml/min of total flow, 0.7 ml/min of column flow and linear velocity of 30.2 cm/s. The oven temperature was programmed as follow: 40 °C for 5 min, increased by 7 °C/min to 220 °C, then the oven was programmed to reach 300 °C at 15 °C/min, this temperature was held for 3 min (total run time: 39 min). The injection port was held at 250 °C.

The mass spectrometer was used with a single quadrupole analyzer in electronic ionization mode, scanned over a mass range of m/z 40–450 amu in the full scan mode. The detector voltage was 0.7 kV. The temperature of interface and ion source were kept constant at 250 °C.

The GC-MS data were analyzed using the section GCMS Post-run Analysis of the GCMS solution software (version 2.4, Shimadzu Corporation). Preliminary identification of compounds was done using both NIST 127 and NIST 147. The identification of the selected list of VOCs was then confirmed spiking the analyzed samples with 0.5 μL of authentic specimens and observing the GC-MS peak overlaps.

From the experimental measurements, 5 replicas for each of the seven kind of samples (a culture media and the sixth cell lines shown in Table 1 have been measured with the GC-MS. Each chromatogram was integrated and peaks were matched and aligned in order to obtain a matrix that contains all the peaks found in the whole set of measurements. Firstly, we did not consider those peaks not above the 1% baseline, as well as those identified as arising from the column and the fiber (siloxanes). From the remaining set of peaks we have used for the following data analysis only those that are present in at least 60% of the total chromatograms. The list of the fourteen selected compounds is shown in Table 2.

### Gas sensor

In this work a commercial metal-oxide semiconductor gas sensor coupled with an original temperature modulation was used.

The sensitivity of metal oxide semiconductor gas sensors is activated at high temperature, it is known that the optimal temperature changes according to the gas at which the sensor is exposed^25^. Then the modulation of the sensor temperature is considered an opportunity to change the selectivity of the sensor, to stabilize the response over long period and to obtain additional information about the gaseous mixtures under test^25^,^26^. To this regard different temporal patters of the temperature has been designed and investigated^25^,^44^,^45^,^46^,^47^. All these experiments shown that the temperature modulation achieves superior performance with respect to the use of a constant temperature. However this approach requires a preliminary optimization of the temperature modulation profile that depends on the VOCs profile of the measured sample. Then the optimization of the temperature profile can be performed only when the composition of the measured samples is a-priori known.

To overcome this limitation we recently introduced a self-adaptive temperature modulation that exploits the gas response of the sensor to drive the working temperature modulation^27^.

Figure 3A shows the concept of the self-adaptive temperature modulation. The output signal of the sensor interface is also used as the input of the electronic circuit driving the temperature modulation. In this way, the signal to a chemical stimulus influences the sensor temperature, giving rise to a specific modulation for each different sample. This concept can be implemented by a variety of circuits. Here we have used a TGS2600 tin oxide gas sensor (Figaro inc.). The sensor interface was a square wave oscillator (Fig. 3B). After a transitory time, the output signal converges to a periodic pattern of pulses (Fig. 3C). If the sample does not change, the sequence of pulses is maintained stable and it can be processed by an asynchronous digital counter (included in the temperature modulation block) giving the driving signal of heater. The number of pulses composing the pattern is twice the modulus of the digital counter. Here the periodic sequence, at the equilibrium, contains 16 pulses (while the counter is a mod-8). Then for each measurement 32 semi-periods of the pulse pattern are extracted as the measurement descriptors and then used in the multivariate analysis. The signal and the corresponding sensor temperature are shown in Fig. 1C. More details about the self-adapted thermal modulation can be found in ref. 27.

The sensor is placed in a chamber of 8 ml of volume while the sample is enclosed in a vial and is headspace is maintained, by means of a thermal bath, at a constant temperature of 37 °C. In order to avoid any condensation problem all the tubes connecting the sample and sensor chamber are maintained at the same temperature. An empty vial placed in the same bath of the culture media samples has been used to have a similar pneumatic path of the measuring phase also for the cleaning step.

The sample headspace is delivered to the sensor by a flow of synthetic air; the same synthetic air is also used as reference to set the sensor baseline (Fig. 3). The sensor was exposed to a constant flow of 40 sccm kept constant by a mass flow controller. For each measurement, the pulse pattern was acquired and the time lengths of the different semi-periods are used as measurement descriptors. Between two consecutive measurements the sensor was flowed with synthetic air for 20 minutes in order to recover the initial condition.

48 measurements have been collected in three measurement sessions along a period of 3 months (6 measures of culture medium, 9 measures of MFC-10A, MDA231 and MCF-7 culture media; six measures for ZR75I and BT474 and three measures for SKBR3).

### Data Analysis

GC-MS data were analyzed using Welch’s t-test and MANOVA to evaluate the statistical difference between the patterns of VOCs related to the cancer cells^48^,^37^.

The gas sensor data (32 semi-periods of the pulse pattern for each measurement) was analyzed with Principal Component Analysis (PCA). Finally the Partial Least Square Discriminant Analysis (PLS-DA) classification model has been used to test the discrimination capabilities of the sensor data to identify the cancer cells or the proliferation rate of the different cell lines (high vs low proliferation)^47^. The performances of the model has been validated with k- fold cross-validation procedure (k = 5). Although the cross-validation procedure gives an overoptimistic estimation of the classification performances, it represent anyway an indication of the potentiality of the proposed approach to identify the different classes.

## Additional Information

**How to cite this article**: Lavra, L. *et al.* Investigation of VOCs associated with different characteristics of breast cancer cells. *Sci. Rep.* **5**, 13246; doi: 10.1038/srep13246 (2015).

## Supplementary Material

Supplementary Information

## Figures and Tables

**Table 1:** Characteristics of breast-derived cell lines used in the study.

Cell line	Isolatedfrom	**Histology**	CDT(h)	**ER**	**PgR**	**HER2**	MolecularSubtype
MCF-10a	BT	FD	96	−	−	−	
MDA-231	PE	ADC	24	−	−	−	*Triple-negative*
MCF-7	PE	IDC	24	+	+	−	*Luminal A*
SKBR-3	PE	ADC	36	−	−	+	*ERB type*
BT-474	PT	IDC	48	−	+	+	*Luminal B*
ZR75-1	PE	IDC	80	+	+	+	*Luminal B*

BT, non-malignant breast tissue; FD, fibrocystic disease; PE, pleural effusion; PT, primary tumor; ADC, adenocarcinoma; IDC, intraductal carcinoma. The results of the IHC characterization of the breast cell lines used in the study are indicated. ER/PgR/HER2 status: ER/PgR positivity, HER2 overexpression. The molecular classification of the breast cancer cell lines subtypes according to WHO guidelines is reported [3].

**Table 2:** VOCs discriminating the headspace of breast-derived cell lines.

**Trend**	**Class**	**Compound**	**CAS number**	**p value**
Increase	Aromatic Amines	Pyrrolidine	123-75-1	0.02
	Hydrocarbons	2,3-Dimethylhexane,	584-94-1	1.8 × 10^−4^
		2,4-Dimethyl-1-heptene	19549-87-2	1.3 × 10^−9^
		2,2-Dimethylbutane	75-83-2	8.1 × 10^−5^
		1,3-Di-tert-butylbenzene	1014-60-4	1.8 × 10^−9^
		2-Xylene	95-47-6	0.77
	Ketons	2-Nonanone	821-55-6	6.5 × 10^−21^
		4-Methyl-2-heptanone	6137-06-0	5.8 × 10^−11^
		2-Dodecanone	6175-49-1	3.6 × 10^−3^
	Carboxylic acid	Isobutyric acid, allyl ester	15727-77-2	3.6 × 10^−10^
	Fatty alcohol	2-Ethylhexanol	104-76-7	0.41
Decrease	Aromatic aldehyde	Benzaldehyde	100-52-7	0.013
	Secondary alcohol	Cyclohexanol	108-93-0	0.92
Manova				1.6 × 10^−16^

Analysis of VOCs in the headspace of breast cell lines by GC-MS. Breast non-transformed and cancer cell lines were cultured at the same conditions. VOCs released (increase) or consumed/degraded (decrease) by breast cell lines are reported with respect to control medium. The p-values obtained by t-test analysis for each compound and by MANOVA analysis for all compounds are shown. A p-value < 0.05 has been considered statistically significant.

**Table 3:** VOCs discriminating breast non-transformed from cancer-derived cell lines.

**Trend**	**Class**	**Compound**	**CAS number**	**p value**
Increase	Hydrocarbons	2,4-Dimethyl-1-heptene	19549-87-2	3.0 × 10^−4^
		2-Xylene	95-47-6	1.0 × 10^−4^
		2,3-Dimethylhexane	584-94-1	1.0 × 10^−4^
		2,2-Dimethylbutane	75-83-2	1.0 × 10^−4^
	Secondary alcohol	Cyclohexanol	108–93-0	2.0 × 10^−5^
	Ketons	2-Dodecanone	6175-49-1	0.003
Decrease	Ketons	2-Nonanone	821-55-6	0.03
		4-Methyl-2-heptanone	6137-06-0	0.04
Manova*				7.7 × 10^−3^

GC-MS analysis in the non-transformed MCF-10A and in cancer derived cell lines. The p-values obtained by t-test analysis for each compound and by MANOVA analysis for all compounds reported in Table 2 are shown. A p-value < 0.05 has been considered statistically significant.

^*^considering all the VOCs of the Table 2.

**Table 4:** Correlation of VOCs with replication time and molecular markers of breast-derived cell lines.

**Class**	**Compound**	**CDT**	**ER status**	**PgR status**	**HER2 status**
		**>48 h vs ≤48 h**	**(−) vs (+)**	**(−) vs (+)**	**(−) vs (+)**
Hydrocarbons	2,4-Dimethyl-1-heptene	2.9 × 10^−4^ 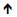	0.78	0.30	0.89
	1,3-Di-tert-butylbenzene	0.29	0.88	0.049 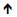	0.25
	2-Xylene	0.14	6.2 × 10^−4^ 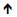	2.5 × 10^−5^ 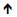	0.08
	2,3-Dimethylhexane	8.4 × 10^−5^ 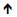	0.98	0.15	0.41
Secondary alcohol	Cyclohexanol	0.001 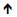	0.42	0.51	0.78
Fatty alcohol	2-Ethylhexanol	1.9 × 10^−6^ 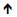	9.1 × 10^−5^ 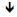	0.046 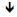	0.16
Carboxylic acid	Isobutyric acid, allyl ester	0.04 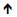	0.12	0.59	0.86
Ketons	2-Dodecanone	0.009 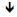	0.003 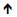	0.005 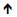	0.01 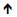
	2-Nonanone	0.11	0.001 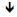	0.58	0.85
	4-Methyl-2-heptanone	0.023 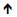	6.4 × 10^−4^ 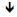	0.18	0.48
Manova		5 × 10^−4^	3.5 × 10^−5^	7.4 × 10^−4^	0.03

Comparison of GC-MS results in all breast-derived cell lines grouped based on CDT, expression of ER, PgR and amplification of HER2. Arrows indicate the trend for each compound in each group. The p-values obtained by t-test analysis for each compound and by MANOVA analysis for all compounds reported in Table 2 are shown. A p-value < 0.05 has been considered statistically significant.

**Table 5:** Confusion Matrix of self-modulation sensor outputs.

		***predicted***	
		**Cancer**	**No cancer**
*measured*	Cancer	29	4
	No cancer	3	12
	*Sensitivity (%)*	88	
	*Specificity (%)*	80	
	*Accuracy (%)*	85	

Classification success of the PLS-DA classification model to predict breast cancer cell lines-derived samples from all other. Sensitivity = TP/(TP + FN); specificity = TN/(TN + FP); accuracy = (TP + TN)/(TP + TN + FP + FN).

Where TP = True Positive; TN = True Negative; FP = False Positive; FN = False Negative.

**Table 6:**
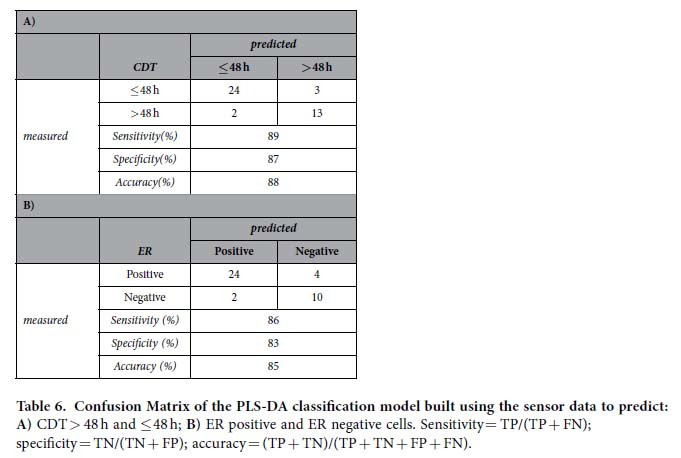
Confusion Matrix of the PLS-DA classification model built using the sensor data to predict:
