# Supplementary material for: Investigation of VOCs associated with different characteristics of breast cancer cells
Source: Sci Rep. 2015 Aug 25;5:13246. doi: 10.1038/srep13246 (PMC4548242; doi:10.1038/srep13246)
Supplement: Supplementary Information [file srep13246-s1.pdf]

Investigation of VOCs associated with different characteristics of breast cancer cells

Authors:

Luca Lavra[1#], Alexandro Catini[2#], Alessandra Ulivieri[1], Rosamaria Capuano[2], Leila Baghernajad Salehi[1], Salvatore Sciacchitano[1,3], Armando Bartolazzi[4,5], Sara Nardis[6], Roberto Paolesse[6], Eugenio Martinelli[2] and Corrado Di Natale[2].*

[1]Biomedical Research Center, "Fondazione Niccolò Cusano per la Ricerca Medico-Scientifica", Niccolò Cusano University, Rome, Italy

[2]Department of Electronic Engineering, University of Rome Tor Vergata, Via del Politecnico 1, 00133 Rome, Italy.

[3]Department of Clinical and Molecular Medicine, University of Rome "Sapienza", Rome, Italy.

[4]Department of Pathology, Universitary Hospital Sant'Andrea, Rome, Italy.

[5]Cellular and Molecular Tumour Pathology Laboratory, Cancer Center Karolinska, Karolinska Hospital, Stockholm, Sweden.

[6]Department of Chemical science and technology, University of Rome Tor Vergata, Via di Tor Vergata, 00133 Rome, Italy.

SUPPLEMENTARY INFORMATION

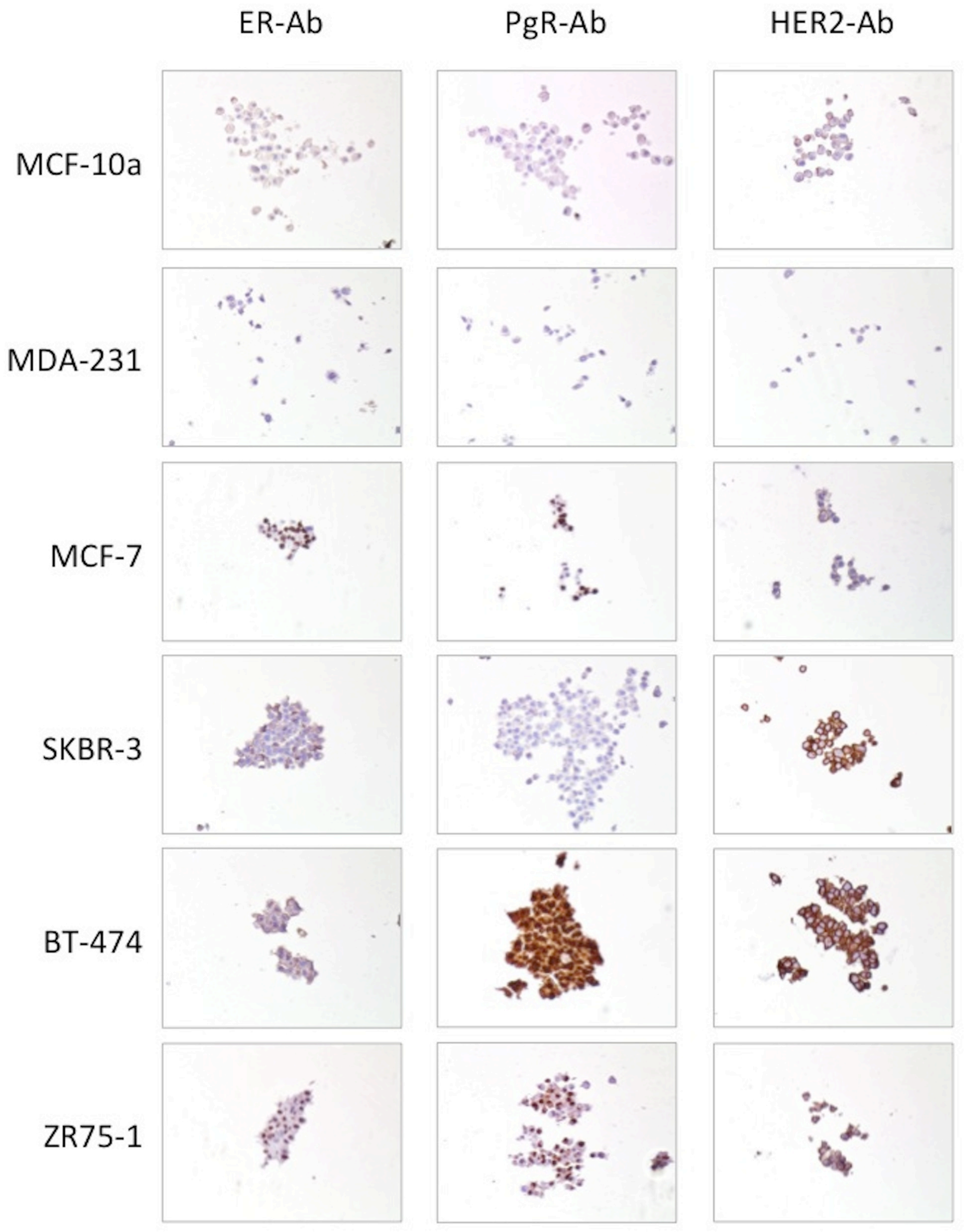

Supplementary Figure 1. Molecular characterization of breast cell lines used in the study

Representative panels of the results of IHC analysis of Estrogen Receptor (ER), Progesteron Receptor (PgR) and HER2 in the indicated cell lines are shown (original magnification 200x). For hormone receptors, cell lines with more than 75% of nuclear immunostaining has been considered positive. For HER2 a complete positivity at the plasma membrane in more than 10% of tumor cells has been considered indicative for overexpression.
